# Cardioprotective Effect of the Mitochondrial Unfolded Protein Response During Chronic Pressure Overload

**DOI:** 10.1016/j.jacc.2018.12.087

**Published:** 2019-04-16

**Authors:** Ioannis Smyrnias, Stephen P. Gray, Darlington O. Okonko, Greta Sawyer, Anna Zoccarato, Norman Catibog, Begoña López, Arantxa González, Susana Ravassa, Javier Díez, Ajay M. Shah

**Affiliations:** a School of Cardiovascular Medicine & Sciences, King’s College London British Heart Foundation Centre, London, United Kingdom; b Program of Cardiovascular Diseases, CIMA, University of Navarra, IdiSNA, Pamplona, Spain, and CIBERCV, Carlos III Institute of Health, Madrid, Spain; c Department of Cardiology and Cardiac Surgery and Department of Nephrology, University of Navarra Clinic, Pamplona, Spain

**Keywords:** cardiomyocyte, heart, mitochondria, pressure overload, unfolded protein response, Atf5, cyclic AMP-dependent transcription factor ATF-5, CHOP, CCAT-enhancer-binding protein homologous protein, ClpP, ATP-dependent Clp protease proteolytic subunit, G-TPP, gamitrinib-triphenylphosphonium, Hsp10, heat shock 10kDa protein 1, Hsp60, heat shock 60kDa protein 1, LonP1, lon protease homolog, mitochondrial, mtDNAj, mitochondrial pre-sequence translocase-associated motor complex protein (PAM16), NR, nicotinamide riboside

## Abstract

**Background:**

The mitochondrial unfolded protein response (UPR^mt^) is activated when misfolded proteins accumulate within mitochondria and leads to increased expression of mitochondrial chaperones and proteases to maintain protein quality and mitochondrial function. Cardiac mitochondria are essential for contractile function and regulation of cell viability, while mitochondrial dysfunction characterizes heart failure. The role of the UPR^mt^ in the heart is unclear.

**Objectives:**

The purpose of this study was to: 1) identify conditions that activate the UPR^mt^ in the heart; and 2) study the relationship among the UPR^mt^, mitochondrial function, and cardiac contractile function.

**Methods:**

Cultured cardiac myocytes were subjected to different stresses in vitro. Mice were subjected to chronic pressure overload. Tissues and blood biomarkers were studied in patients with aortic stenosis.

**Results:**

Diverse neurohumoral or mitochondrial stresses transiently induced the UPR^mt^ in cultured cardiomyocytes. The UPR^mt^ was also induced in the hearts of mice subjected to chronic hemodynamic overload. Boosting the UPR^mt^ with nicotinamide riboside (which augments NAD^+^ pools) in cardiomyocytes in vitro or hearts in vivo significantly mitigated the reductions in mitochondrial oxygen consumption induced by these stresses. In mice subjected to pressure overload, nicotinamide riboside reduced cardiomyocyte death and contractile dysfunction. Myocardial tissue from patients with aortic stenosis also showed evidence of UPR^mt^ activation, which correlated with reduced tissue cardiomyocyte death and fibrosis and lower plasma levels of biomarkers of cardiac damage (high-sensitivity troponin T) and dysfunction (N-terminal pro–B-type natriuretic peptide).

**Conclusions:**

These results identify the induction of the UPR^mt^ in the mammalian (including human) heart exposed to pathological stresses. Enhancement of the UPR^mt^ ameliorates mitochondrial and contractile dysfunction, suggesting that it may serve an important protective role in the stressed heart.

The mammalian heart has the highest oxygen consumption rate of any organ, which may transiently increase several-fold during physiological exercise to support the increased cardiac workload. Chronic increases in cardiac workload resulting from hemodynamic overload or neurohumoral activation eventually lead to heart failure. Approximately 26 million patients worldwide are estimated to have heart failure, and the condition imposes substantial morbidity and mortality despite the use of therapies that target neurohumoral activation and cardiac rhythm disturbance (1).

Mitochondria are crucial for cardiac function through oxidative ATP generation to support muscle contraction and relaxation, the metabolism of nucleotides, amino acids and lipids, intracellular calcium buffering, and the regulation of cardiomyocyte survival. Mitochondrial dysfunction is a central feature of heart failure by contributing to energetic dysfunction, oxidative stress, calcium dysregulation, and cardiomyocyte death, and is considered a potential therapeutic target (2). The vast majority of the >1,000 mitochondrial proteins are encoded by nuclear genes and are imported. Both mitochondrial-encoded and nuclear-encoded proteins undergo folding and/or assembly into multimolecular complexes within mitochondria (3). Under cellular stress conditions, the mitochondrial protein-folding environment may be challenged and result in the production of misfolded, dysfunctional proteins. Dysfunction of the mitochondrial respiratory chain is accompanied by increased generation of reactive oxygen species (ROS) and oxidative stress, which in turn compromises protein integrity and folding—resulting in a vicious cycle of progressively worsening mitochondrial damage and heart dysfunction (2).

The mitochondrial unfolded protein response (UPR^mt^) is evolutionarily conserved and evoked when the mitochondrial protein folding environment is compromised and there is an accumulation of misfolded proteins (4). The UPR^mt^ is a mitochondrial-to-nuclear signal transduction pathway that leads to increased transcription of numerous mitochondrial protective genes, notably a repertoire of molecular chaperones, proteases, and antioxidant enzymes located primarily in the mitochondrial matrix (5). These include chaperones (e.g., mitochondrial pre-sequence translocase-associated motor complex protein [mtDNAj], heat shock 10kDa protein 1 [Hsp10], heat shock 60kDa protein 1 [Hsp60]), which facilitate protein folding or repair misfolded proteins, and proteases (e.g., ATP-dependent Clp protease proteolytic subunit [ClpP], lon protease homolog, mitochondrial [LonP1], Htra2), which degrade unrepairable and/or truncated proteins for efficient removal. Activation of the UPR^mt^ serves to restore mitochondrial protein homeostasis, function, and cell survival. The UPR^mt^ has been extensively characterized in *C. elegans* 6, 7 and demonstrated in yeast (8), flies (9), and mammalian cells (10), and has been associated with potential cytoprotective roles in diverse models of stress 4, 11, 12. However, the role of the UPR^mt^ in the heart is unclear. Here, we identify the induction of the UPR^mt^ in cardiomyocytes subjected to neurohumoral or mitochondrial stress and in the hemodynamically overloaded murine and human heart. We show that pharmacological enhancement of UPR^mt^ with small-molecule agents ameliorates mitochondrial and contractile dysfunction in the overloaded murine heart, and in patients with aortic stenosis, elevated levels of UPR^mt^ indexes correlate with reduced plasma biomarkers of cardiac damage and dysfunction. The UPR^mt^, therefore, has an important homeostatic role in the pathologically stressed mammalian heart and may be a potential therapeutic target in heart failure.

## Methods

Detailed methods appear in the Online Appendix. Animal procedures were performed in accordance with the United Kingdom’s legal and institutional ethical guidelines. Male mice were subjected to transverse aortic constriction (TAC) or a sham procedure under isoflurane anesthesia. Oxygen consumption of neonatal rat cardiomyocyte cultures was quantified on an extracellular flux analyzer (Seahorse, Agilent Technologies, Santa Clara, California), according to the manufacturer’s protocol for mitochondrial stress tests. Data were normalized by cell number. Tissue myocardial oxygen consumption rate was measured in an Oxytherm (Hansatech, King's Lynn, United Kingdom). mRNA levels were quantified by real-time PCR using SYBR Green and the comparative Ct method, with cytoskeletal β-actin levels used for normalization. Primer sequences are shown in Online Table 1. Immunoblotting was performed using standard methods. Histology of mouse hearts was performed in paraformaldehyde-fixed samples. Cyclic AMP-dependent transcription factor ATF-5 (Atf5) silencing was performed in neonatal cardiomyocytes using TransFectin-mediated siRNA transfection.

Human studies were performed under institutional ethical approval and with informed consent. Patients undergoing valve replacement for severe aortic stenosis were included. Echocardiography was performed pre-operatively, and blood samples were obtained for biomarkers. Myocardial biopsies were obtained during surgery and used for mRNA analyses and histology. Control samples were obtained from subjects who had died from noncardiovascular causes.

Data are presented as mean ± SEM, unless otherwise stated. Comparisons were made by Student’s *t*-test, Mann-Whitney *U* test, 1-way analysis of variance (ANOVA), 2-way ANOVA, or repeated measures ANOVA as appropriate, followed by Bonferroni post-hoc testing. A p value <0.05 was considered significant.

## Results

### UPR^mt^ induction in cardiomyocytes subjected to pathological stresses and in the hemodynamically overloaded heart

**Figure 1:**
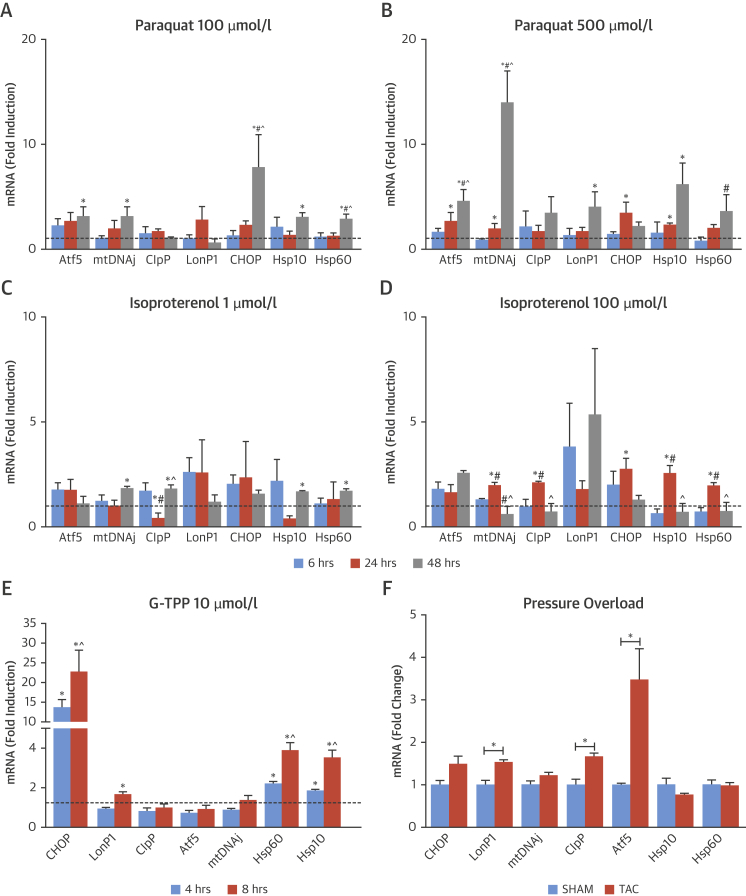
Induction of UPR^mt^ Markers Under Various Stress Conditions in Cardiomyocytes and in an In Vivo Model of Chronic Pressure Overload **(A and B)** Response of cardiomyocytes to paraquat (100 or 500 μmol/l for 6, 24, and 48 h). **(C and D)** Response of cardiomyocytes to isoproterenol (1 or 100 μmol/l for 6, 24, and 48 h). **(E)** Response of cardiomyocytes to G-TPP (10 μmol/l for 4 and 8 h). *p < 0.05 versus respective control for changes in mRNA levels. #p < 0.05 versus 6-h treatment. ˆp < 0.05 versus 24-h treatment. **(F)** Effect of chronic pressure overload (TAC) on UPR^mt^ markers. *p < 0.05 versus sham-operated control mice or control conditions in NRVM. **Dashed line** denotes mRNA levels under control conditions. Data are mean ± SEM, n = 4 to 8/group. Atf5 = cyclic AMP-dependent transcription factor ATF-5; CHOP = CCAT-enhancer-binding protein homologous protein; ClpP = ATP-dependent Clp protease proteolytic subunit; G-TPP = gamitrinib-triphenylphosphonium; Hsp10 = Heat shock 10kDa protein 1; Hsp60 = Heat shock 60kDa protein 1; LonP1 = Lon protease homolog, mitochondrial; mtDNAj = mitochondrial pre-sequence translocase-associated motor complex protein; UPR^mt^ = mitochondrial unfolded protein response.

The induction of the UPR^mt^ involves the transcription factors Atf5 and CCAT-enhancer-binding protein homologous protein (CHOP), which increase the expression of mitochondrial chaperones and proteases 10, 13. We first tested the effect of subjecting cultured rat cardiomyocytes to 4 different pathological stresses: 1) mitochondrial stress by treatment with the complex I inhibitor paraquat; 2) neurohumoral stress by treatment with the β-adrenoreceptor agonist isoproterenol (Iso); 3) mitochondrial stress by treatment with an inhibitor of the mitochondrial chaperone Hsp90, Gamitrinib-triphenylphosphonium (G-TPP); and 4) mitochondrial protein misfolding stress by the overexpression of a terminally misfolded mutant form of mitochondrial ornithine transcarbamylase (Δ-OTC) (10). Cardiomyocytes treated with incremental concentrations of paraquat or isoproterenol showed significant time-dependent increases in the mRNA levels of Atf5, CHOP, mtDNAj, ClpP, LonP1, Hsp10, and Hsp60 (Figures 1A to 1D). The increases in mRNA levels of the mitochondrial chaperones and proteases were either transient (only at earlier time points after Iso) or only occurred at later time points (e.g., after paraquat). Treatment of cardiomyocytes with G-TPP (10 μmol/l) resulted in significant increases in the mRNA levels of CHOP, LonP1, Hsp60, and Hsp10 after either 4 or 8 h of treatment (Figure 1E). Finally, the overexpression of Δ-OTC also resulted in significant elevation in the mRNA levels of Atf5, CHOP, mtDNAj, ClpP, and LonP1 in a similar range to that reported previously in other systems 10, 13 (Online Figure 1A).

To assess whether UPR^mt^ induction in cardiomyocytes occurs as a process distinct from the endoplasmic reticulum unfolded protein response (ER stress) or cytosolic stress responses, we assessed the protein levels of KDEL sequence-containing markers of ER stress (i.e., calreticulin, Grp78, and Grp79) and mRNA levels of the cytosolic chaperones Hsp70 and Hsp90 under these stress conditions. Protein levels of KDEL sequence-containing proteins were unaltered in cardiomyocytes treated with isoproterenol, paraquat, or Δ-OTC overexpression, whereas stimulation with tunicamycin, which induces ER stress, robustly increased the levels of these proteins (Online Figure 1). mRNA levels of cytosolic Hsp70 and Hsp90 were also largely unaltered by the UPR^mt^-inducing stress stimuli (Online Figure 1).

We next investigated whether the UPR^mt^ is activated during in vivo pathological cardiac stress induced by TAC. Analysis of LV tissue 14 days after TAC showed a significant up-regulation in mRNA levels of Atf5, ClpP, and LonP1, whereas levels of CHOP, mtDNAj, and Hsp10/60 remained unaltered (Figure 1F). TAC resulted in significant cardiac hypertrophy (heart/body weight ratio 6.32 ± 0.34 vs. 4.30 ± 0.11 in SHAM; p < 0.05) and LV contractile impairment (ejection fraction 26.8 ± 2.1% vs. 62.7 ± 2.8% in SHAM; p < 0.05).

These data demonstrate for the first time that the UPR^mt^ is induced in cardiomyocytes subjected to diverse pathological stresses and in the hemodynamically overloaded heart in vivo.

### Agents that enhance NAD^+^ levels boost UPR^mt^ markers in cardiomyocytes and the heart in vivo

**Figure 2:**
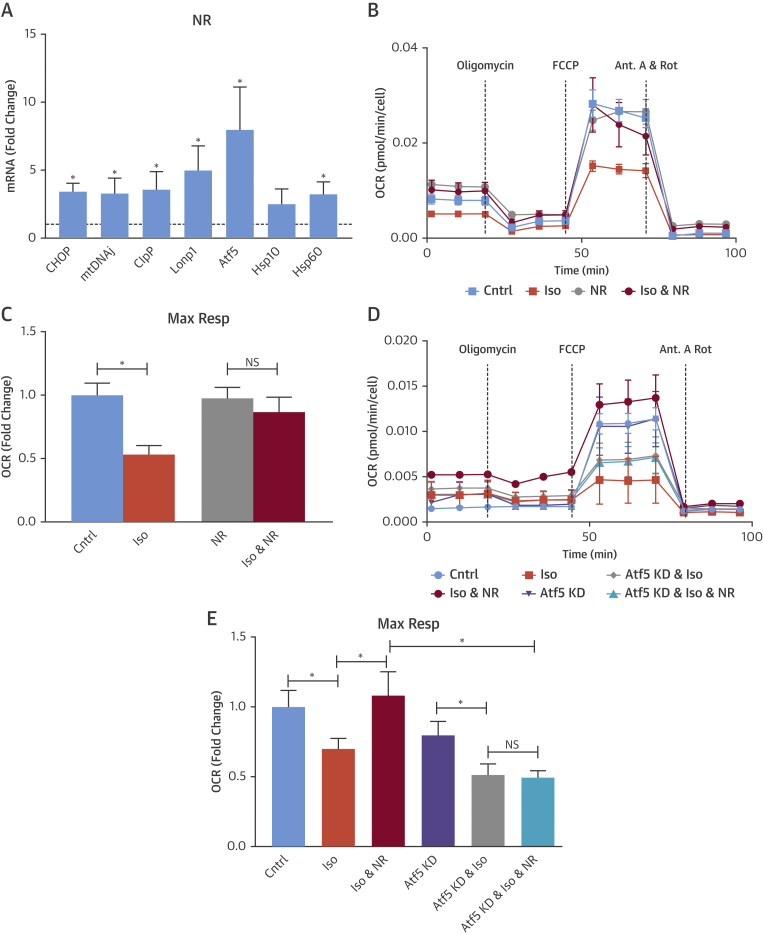
Enhancement of UPR^mt^ Prevents Stress-Induced Decrease in Mitochondrial Maximum Respiration Rate (Max Resp) **(A to E)** Cardiomyocytes treated with NR (1 mmol/l) demonstrated increased mRNA levels of UPR^mt^ markers **(A)**. Representative traces **(B)** and averaged data **(C)** showing that enhancement of UPR^mt^ with NR ameliorated the isoproterenol-induced decrease in maximum respiration rate (Max Resp). Representative traces **(D)** and averaged data **(E)** showing that the amelioration of isoproterenol-induced decrease in maximum respiration rate by NR is prevented in the setting of Atf5 knockdown. *p < 0.05 versus untreated control represented by **dashed line**; #p < 0.05 versus 6-h NR treatment. Data are mean ± SEM, n = 5 to 7/group. Cntrl = control; Iso = isoproterenol; NR = nicotinamide riboside; other abbreviations as in Figure 1.

Previous studies showed that the enhancement of cellular NAD^+^ pools, either directly via supplementation with nicotinamide riboside (NR) or indirectly via the pharmacological inhibition of polyADP-ribose polymerase (PARP), boosts the UPR^mt^ 12, 14, 15, 16. Consistent with these data, treatment of cardiomyocytes with NR (1 mmol/l for 18 h) significantly increased the mRNA levels of Atf5, CHOP, mtDNAj, ClpP, Lonp1, and Hsp60 (Figure 2A), whereas cytosolic Hsp70 and Hsp90 levels were not increased (Online Figure 2A). Likewise, the treatment of cardiomyocytes with the PARP inhibitor olaparib (10 μmol/l for 24 to 48 h) caused a significant increase in the mRNA levels of Atf5, mtDNAj, ClpP, Lonp1, Hsp10, and Hsp60 while not affecting Hsp70 and Hsp90 levels (Online Figures 2B and 2C). The in vivo treatment of mice with NR (750 mg/kg/day intraperitoneally for 3 days) also significantly upregulated the mRNA levels of Atf5, CHOP, ClpP, and mtDNAj compared with control vehicle-treated mice (Online Table 2).

### Pharmacological enhancement of the UPR^mt^ rescues mitochondrial dysfunction in stressed cardiomyocytes and in the overloaded heart in vivo

To determine the functional effect of boosting the UPR^mt^ in stressed cardiomyocytes, we compared the effects of Iso (100 μmol/l, 24 h) stimulation on mitochondrial respiration with and without pre-treatment with NR or olaparib to enhance the UPR^mt^. Iso stimulation resulted in a significant decrease in mitochondrial maximal respiration, which was markedly improved when the UPR^mt^ was boosted with NR (Figures 2B and 2C) or olaparib (Online Figures 2C and 2D).

Atf5 is reported to be a key transcriptional inducer of the mammalian UPR^mt^ 13, 17. Indeed, silencing of Atf5 in cardiomyocytes abolished the Iso-induced increase in UPR^mt^ markers (Online Figure 3A). To confirm that the effects of NR are mediated at least in part through activation of the UPR^mt^, we silenced Atf5 and then examined the effects of NR on UPR^mt^ markers. The silencing of Atf5 substantially inhibited the NR-mediated increase in UPR^mt^ markers (Online Figure 3B) and abolished the NR-mediated prevention of Iso-induced decrease in mitochondrial respiration (Figures 2D and 2E), strongly supporting the idea that NR is protective by boosting the UPR^mt^.

**Figure 3:**
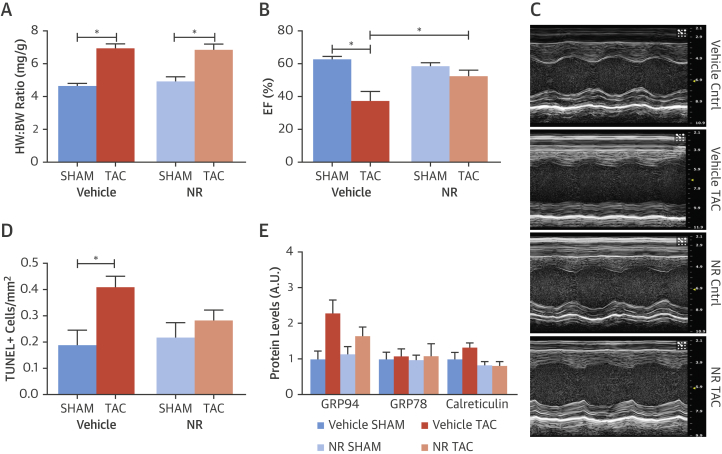
Enhancement of UPR^mt^ With NR Is Cardioprotective in Mice Subjected to Chronic Pressure Overload Mice were pre-treated with NR or vehicle for 3 days and subsequently subjected to TAC or sham-constriction. **(A)** Heart weight/body weight (HW:BW) ratio as a measure of cardiac hypertrophy. **(B)** Effect of NR on left ventricular ejection fraction (EF). **(C)** Representative M-mode echocardiography images. **(D)** Quantification of TUNEL^+^ cardiomyocytes in myocardial sections. **(E)** Changes in KDEL sequence-containing proteins assessed by immunoblotting. Data are mean ± SEM, n = 4 to 14/group; *p < 0.05. TAC = transverse aortic constriction; other abbreviations as in Figures 1 and 2.

We next tested the effects of boosting the UPR^mt^ in vivo on cardiac functional responses to TAC-induced chronic pressure overload. Mice were treated with NR or control vehicle for 3 days before subjecting them to TAC. The extent of hypertrophy induced by TAC was similar in NR- and vehicle-treated mice (Figure 3A). However, LV function assessed by the echocardiographic ejection fraction 1 week after TAC was significantly better in NR-treated compared with vehicle-treated mice (Figures 3B and 3C). Histological analyses of myocardial tissue from these animals revealed that NR-treated animals had a significantly lower number of TUNEL^+^ cardiomyocytes after TAC than vehicle-treated mice subjected to TAC (Figure 3D). Treatment with NR did not affect the ER stress response (Figure 3E, Online Figure 4).

**Figure 4:**
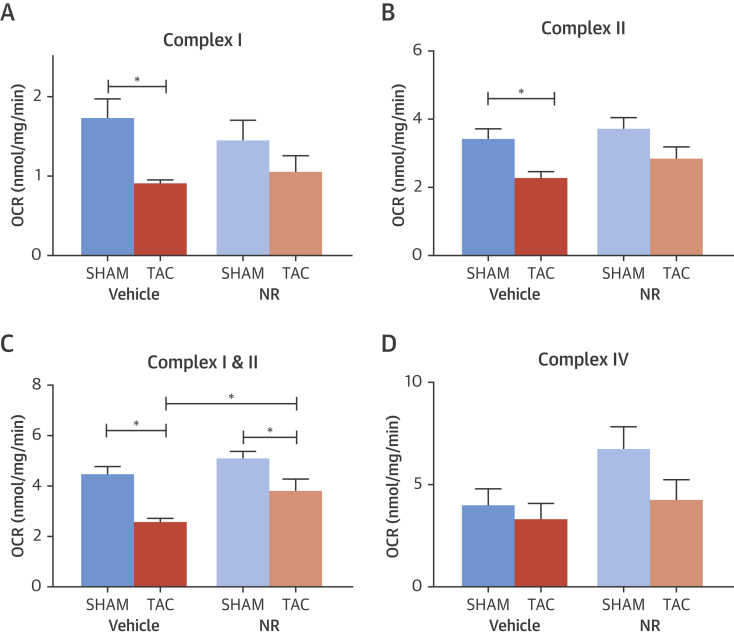
Effect of Boosting the UPR^mt^ on Myocardial Mitochondrial Respiration in Mice Subjected to TAC or SHAM **(A to D)** Oxygen consumption rate (OCR) in ventricular muscle for Complex I, Complex II, Complex I + II, and Complex IV. Mice were pre-treated with NR or vehicle and then subjected to TAC or sham. Data are mean ± SEM, n = 4 to 6/group; *p < 0.05. NR = nicotinamide riboside; TAC = transverse aortic constriction.

We assessed the effect of NR pre-treatment on mitochondrial respiration in myocardial tissue from NR- and vehicle-treated mice subjected to TAC or sham surgery. TAC resulted in a reduced mitochondrial complex I- and complex II-dependent oxygen consumption rate as compared to sham (Figures 4A to 4C). This impairment was significantly improved in mice pre-treated with NR to induce the UPR^mt^. No differences were detected in complex IV-dependent oxygen consumption rate (Figure 4D). These data demonstrate that pharmacological boosting of the UPR^mt^ improves mitochondrial function, cardiomyocyte survival, and contractile function in animals subjected to chronic hemodynamic overload.

### Induction of the UPR^mt^ in the hearts of humans with chronic pressure overload

**Figure 5:**
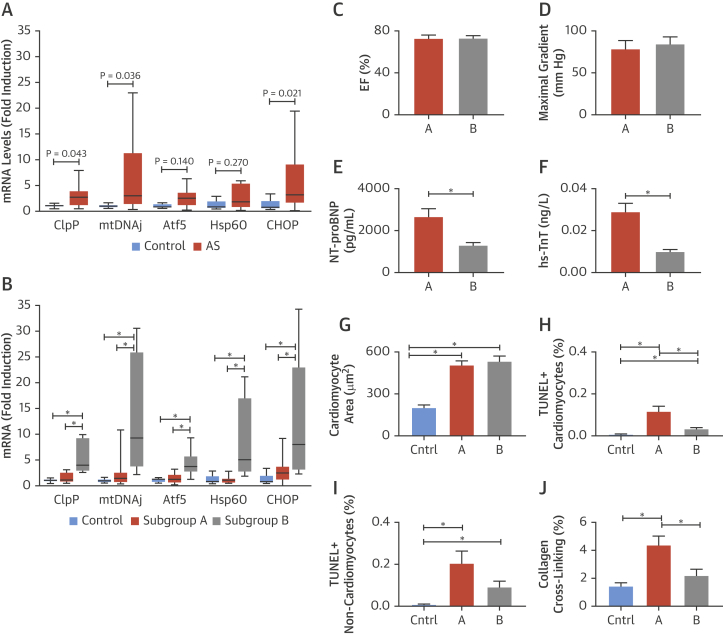
UPR^mt^ Markers in Human Myocardium From AS Patients Undergoing Valve Replacement Surgery or Control Subjects **(A)** mRNA levels of UPR^mt^ markers in myocardium from aortic stenosis (AS) patients undergoing valve replacement surgery compared with control myocardium. **(B)** Patients were divided into 2 subgroups based on the mRNA levels of UPR^mt^ markers relative to the upper limit of normality (2× the SD of control measurements for each respective target). mRNA levels of ClpP, mtDNAj, Atf5, Hsp60, and CHOP are shown. Data in **A and B** are expressed as median ± 95th percentile, n = 5 to 15/group; *p < 0.05. **(C to J)** Comparison of clinical and histological characteristics between AS patients with UPR^mt^ markers in the normal range (subgroup A) and those with elevated UPR^mt^ markers (subgroup B). Left ventricular ejection fraction (EF) by echocardiography **(C)**; maximal aortic transvalvular pressure gradient **(D)**; serum levels of N-terminal pro–B-type natriuretic peptide (NT-proBNP) **(E)**; serum levels of high-sensitivity troponin T (hsTnT) **(F);** extent of cardiomyocyte hypertrophy **(G)**; TUNEL^+^-cardiomyocytes **(H)** and TUNEL^+^-noncardiomyocytes in myocardial sections **(I)**; and myocardial collagen cross-linking **(J).** Data are mean ± SEM, n = 5 to 15/group. *p < 0.05. Abbreviations as in Figure 1.

To investigate the relevance of the UPR^mt^ in the pathologically stressed human heart, we analyzed myocardial tissue from patients who underwent valve replacement surgery for severe aortic stenosis (AS), a clinical model of chronic pressure overload. The clinical and demographic characteristics of these patients are shown in Online Table 3. Control myocardial samples were obtained from subjects who had died from trauma or neurological injury. Gene expression analyses revealed a significant increase in the mRNA levels of ClpP, mtDNAj, and CHOP in the samples from AS subjects compared with control subjects (Figure 5A). However, we also noted that there was a wide variation in the mRNA levels of UPR^mt^ markers among the AS group compared with control. Notably, approximately 50% of the AS subjects had substantial increases in the expression levels of these transcripts (Figure 5A).

To elucidate any relationship between the level of increase in myocardial UPR^mt^ markers and patient characteristics, we divided the AS patients into 2 groups (subgroups A and B) based on their myocardial mRNA levels of UPR^mt^ markers being higher or lower than the upper limit of “normality” (ULN). ULN was set as 2 SDs above the mean for each transcript in the control group. Subgroup A comprised AS patients with mRNA levels of UPR^mt^ markers within the normal range, and subgroup B comprised those with mRNA levels of at least 3 UPR^mt^ markers above the upper limit. Subjects in subgroup B in fact demonstrated significant elevation of all the UPR^mt^ markers compared with subjects in subgroup A or control, indicating a significant activation of UPR^mt^ in this cohort (Figure 5B).

We compared a range of parameters of clinical AS severity, hemodynamic state, cardiac structure and function, myocardial histology, and blood biomarkers that were assessed pre-operatively between the 2 subgroups. There was no significant difference between subgroups in age, sex, comorbidities, systemic blood pressure or AS severity, as assessed by the mean transvalvular pressure gradient at echocardiography (Online Table 3). Fewer patients had a history of heart failure in subgroup B than A but this was not significant. The 2 subgroups had similar maximal transvalvular pressure gradients, similar LV systolic contractile function as assessed by ejection fraction, similar LV end-systolic and -diastolic dimensions, and a similar extent of hypertrophy as assessed by the LV mass index or the mean cardiomyocyte area on myocardial sections (Figure 5, Online Table 3). There were no significant differences between subgroups in LV diastolic function as assessed by the Doppler E/A ratio or mitral E-wave deceleration time. However, histological analysis of myocardial sections revealed that subgroup B had significantly lower numbers of TUNEL-positive cardiomyocytes and a lower degree of interstitial collagen cross-linking as an index of myocardial fibrosis than subgroup A (Figure 5). Furthermore, patients in subgroup B had significantly lower serum levels of both hs-TnT and N-terminal pro–B-type natriuretic peptide (Figure 5).

Taken together, these data demonstrate that the UPR^mt^ is induced in the human heart subjected to chronic pathological pressure overload and that a high level of activation is associated with reduced cardiomyocyte cell death, which may consequently limit pathological fibrosis and overall pathological cardiac stress.

## Discussion

Numerous cellular processes depend upon normal mitochondrial function, which is required for cellular metabolism, energy generation, and the regulation of cell death. The mitochondrial proteome may be markedly affected by stress conditions that alter the protein-folding environment 3, 4. Well-characterized, evolutionarily conserved stress responses are evoked in several cellular compartments in response to stresses that disrupt protein integrity, for example, the ER stress response (18), the nucleolar stress response (19), and the cytosolic heat shock response (20). These responses typically lead to an increased expression of proteins that restore organelle homeostasis. The UPR^mt^ was relatively recently identified and serves an analogous function in the mitochondria. While it has been comprehensively characterized in *C. elegans* 6, 7 and identified in other organisms such as yeast (8) and flies (9), it has not been extensively studied in mammals. In particular, the role of the UPR^mt^ in the heart—the organ with the highest oxygen consumption rate in mammals—is unclear. This study identifies an important role for the UPR^mt^ in cardiac stress responses, especially heart failure, where enhancement of the UPR^mt^ substantially attenuates mitochondrial dysfunction and contractile failure in the experimental setting (Central Illustration).

**Central Illustration:**
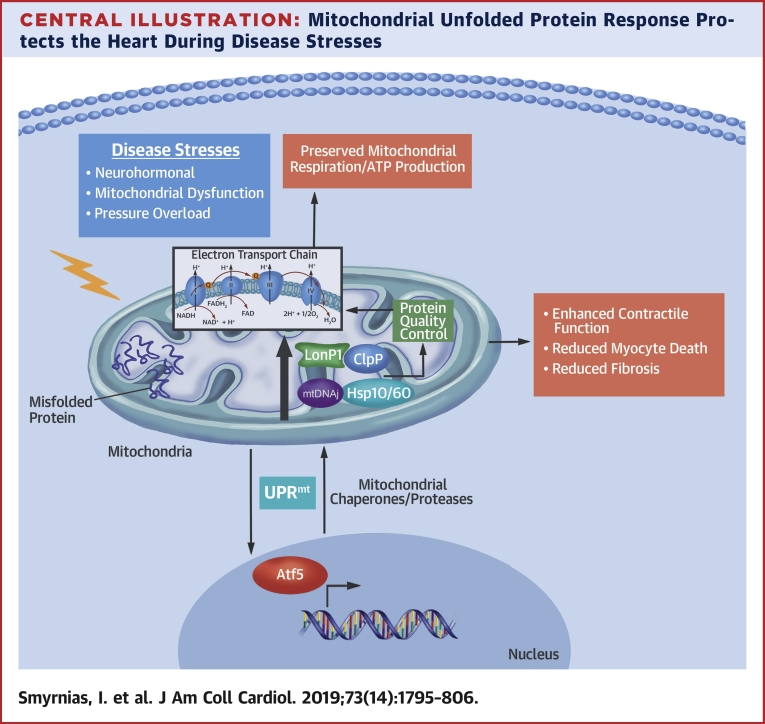
Mitochondrial Unfolded Protein Response Protects the Heart During Disease Stresses Diverse disease stresses induce the mitochondrial unfolded protein response (UPR^mt^) in cardiomyocytes, which leads to an increased expression of mitochondrial chaperones and proteases (e.g., LonP1, ClpP, mtDNAj, Hsp10, Hsp 60). These enhance protein quality control and mitochondrial respiration, thereby enhancing contractile function and reducing myocyte cell death and fibrosis. Atf5 = cyclic AMP-dependent transcription factor ATF-5; ClpP = ATP-dependent Clp protease proteolytic subunit; FAD = oxidized flavin adenine dinucleotide; FADH = reduced flavin adenine dinucleotide; H = hydrogen; H_2_O = water; Hsp10 = Heat shock 10kDa protein 1; Hsp60 = Heat shock 60kDa protein 1; LonP1 = Lon protease homolog, mitochondrial; mtDNAj = mitochondrial pre-sequence translocase-associated motor complex protein; NAD = oxidized nicotinamide adenine dinucleotide; NADH = reduced nicotinamide adenine dinucleotide; O_2_ = oxygen.

Diverse in vitro stresses, such as neurohumoral activation and mitochondrial stress, evoked by the inhibition of complex I by paraquat, inhibition of the mitochondrial chaperone Hsp90 by G-TPP, or excess unfolded proteins (resulting from the overexpression of Δ-OTC), activated the UPR^mt^ in cultured mammalian cardiomyocytes—as evidenced by the increased mRNA expression of several mitochondrial chaperones and proteases and the transcription factors Atf5 and/or CHOP 10, 13. A similar activation of the cardiac UPR^mt^ was observed after the imposition of in vivo hemodynamic overload in the mouse. The level of increase in mRNA levels of UPR^mt^ markers was in a similar dynamic range to that reported after genetic UPR^mt^ induction in *C. elegans* (21). There were some variations between stressors in the genes that were up-regulated among Atf5, CHOP, mtDNAj, ClpP, LonP1, Hsp10, and Hsp60, which may reflect differences in the precise regulation of individual genes (22). Atf5, similarly to ATFS-1, which mediates the UPR^mt^ in *C. elegans* (6), is a bZIP transcription factor that shuttles from the mitochondria to the nucleus during mitochondrial stress and has been reported as 1 of the key mediators of the mammalian UPR^mt^ 10, 13. Indeed, we show that silencing of Atf5 in cardiomyocytes blunts the Iso-induced increase in UPR^mt^ markers. CHOP, on the other hand, is best known to be associated with the ER stress response (18), but several studies (including the current study) found a specific involvement of CHOP in the UPR^mt^ independent of ER stress 5, 10, 23. We found that the activation of the UPR^mt^ occurred distinct from ER stress or the cytosolic heat shock response as evidenced by the lack of corresponding changes in ER stress markers or cytosolic heat shock proteins (Online Figure 1). We used the levels of KDEL sequence-containing proteins as an integrated readout of the ER stress response rather than dissecting individual limbs of this pathway. It may be of interest in future studies to look for any inter-relationship between Atf5 and individual limbs of the ER stress response, including ER stress-related transcription factors (e.g., Atf6), and whether these have any role in the UPR^mt^. Further studies are also required to define the precise inter-relationship during UPR^mt^ activation between Atf5 and CHOP, which is normally linked to activation of the ER stress response.

Activation of the UPR^mt^ has been viewed as a cytoprotective response that promotes organelle recovery 6, 24. In our cardiomyocyte studies, we found that induction of UPR^mt^ markers was typically transient. We therefore hypothesized that enhancing the UPR^mt^ might elicit cytoprotective effects. Recent studies have identified agents that activate the UPR^mt^, thereby facilitating characterization of its functional impact under stress conditions. Notably, the augmentation of cellular NAD^+^ pools leads to the induction of the UPR^mt^, secondary to activation of NAD^+^-dependent deacetylases, and was shown to improve the outcome under conditions of mitochondrial stress^12^, ^14^, ^15^, ^16^. Augmentation of NAD^+^ pools can be achieved via dietary supplementation with NAD^+^ precursors such as NR, or through the inhibition of NAD^+^-consuming enzymes such as PARP. We confirmed that agents that augment the NAD^+^ pools, in particular NR, enhance the UPR^mt^ in cardiomyocytes and that this occurs through the actions of Atf5. We found that the augmentation of the UPR^mt^ led to significant improvement in mitochondrial dysfunction in stressed cardiomyocytes. Importantly, these effects were dependent on Atf5, indicating that they were likely to involve the UPR^mt^ rather than other NAD^+^-dependent actions in the cell. To extend these data to the in vivo setting, we tested the effects of NR supplementation in mice subjected to TAC. We found that NR treatment markedly improved mitochondrial respiration, cardiomyocyte survival, and cardiac contractile function. These data provide proof-of-concept that enhancement of the UPR^mt^ in the pathologically stressed heart may have therapeutic potential.

To extend these findings to the human setting, we studied myocardial tissue from patients with severe AS undergoing valve replacement. A comprehensive molecular and histological analysis of myocardial tissue, clinical characteristics, and blood biomarkers revealed a striking relationship between enhanced activation of the UPR^mt^ and reduced indexes of ongoing myocardial damage. We found that AS patients who had an elevation of UPR^mt^ markers had lower plasma levels of hs-TnT and N-terminal pro–B-type natriuretic peptide, both strong predictors of future adverse outcome independent of the resting LV function (25). They also showed lower levels of apoptotic cardiomyocytes in myocardial sections and less abnormal fibrosis than patients with low levels of UPR^mt^ markers. These findings are consistent with the postulate from our experimental studies that increased activation of the UPR^mt^ is beneficial by preserving mitochondrial function. In the human AS setting, a chronic improvement in mitochondrial function may manifest as a lower rate of cardiomyocyte death, with concomitant lower levels of fibrosis in the myocardium. Unlike the TAC setting, AS subjects with elevated UPR^mt^ markers had similar cardiac function to those with low UPR^mt^ levels, which may reflect the very slowly-progressive pathology of human AS compared with surgically induced TAC in mouse models (25). These data identify for the first time an activation of the UPR^mt^ in the pressure-overloaded human heart and suggest that increased activation of the UPR^mt^ may be beneficial.

Activation of the ER stress response has been demonstrated to be an important pathophysiological mechanism in many human diseases (18) and small molecules that modulate this response are under investigation as potential therapeutic agents (26). Here, we show that the augmentation of a different stress response, the UPR^mt^, may also have beneficial effects. It should be noted, however, that prolonged or dysregulated induction of the UPR^mt^ could be detrimental, for example, contributing to an accumulation of defective mitochondria (27) and neurodegenerative phenotypes (28).

### Study limitations

Future studies will need to investigate the effects of different durations of UPR^mt^ activation and in different disease settings to better understand the therapeutic potential and/or toxicity of such interventions. Another limitation of our study is the fact that the human data are by necessity correlative and that the control subjects were not perfectly matched with the AS patients. However, there was a very good matching of most clinical characteristics between AS patients in the two subgroups.

## Conclusions

In summary, this is the first study to identify activation of the UPR^mt^ in response to diverse in vitro and in vivo stresses in the mammalian myocardium. We also show that pharmacological boosting of the UPR^mt^ preserves mitochondrial function and cardiomyocyte viability under pathological stress conditions relevant to heart failure. Taken together, the results suggest that the targeting of the UPR^mt^ merits further investigation as a therapeutic strategy for heart failure.

Perspectives **COMPETENCY IN MEDICAL KNOWLEDGE:** The detrimental consequences of chronic hemodynamic overload or neurohumoral stress in the heart include mitochondrial dysfunction and contractile depression. Activation of the UPR^mt^ is able to mitigate these effects by restoring mitochondrial homeostasis.

**TRANSLATIONAL OUTLOOK:** The UPR^mt^ can be activated by small molecules that increase NAD^+^ levels. Additional studies are required to determine whether long-term enhancement of the UPR^mt^ is beneficial in chronic hemodynamic overload.

## References

[1] Ambrosy A.P., Fonarow G.C., Butler J. (2014). The global health and economic burden of hospitalizations for heart failure: lessons learned from hospitalized heart failure registries. J Am Coll Cardiol.

[2] Dietl A., Maack C. (2017). Targeting mitochondrial calcium handling and reactive oxygen species in heart failure. Curr Heart Fail Rep.

[3] Voos W., Jaworek W., Wilkening A., Bruderek M. (2016). Protein quality control at the mitochondrion. Essays Biochem.

[4] Haynes C.M., Ron D. (2010). The mitochondrial UPR - protecting organelle protein homeostasis. J Cell Sci.

[5] Pellegrino M.W., Nargund A.M., Haynes C.M. (2013). Signaling the mitochondrial unfolded protein response. Biochim Biophys Acta.

[6] Nargund A.M., Pellegrino M.W., Fiorese C.J., Baker B.M., Haynes C.M. (2012). Mitochondrial import efficiency of ATFS-1 regulates mitochondrial UPR activation. Science.

[7] Yoneda T., Benedetti C., Urano F., Clark S.G., Harding H.P., Ron D. (2004). Compartment-specific perturbation of protein handling activates genes encoding mitochondrial chaperones. J Cell Sci.

[8] Schleit J., Johnson S.C., Bennett C.F. (2013). Molecular mechanisms underlying genotype-dependent responses to dietary restriction. Aging Cell.

[9] Owusu-Ansah E., Song W., Perrimon N. (2013). Muscle mitohormesis promotes longevity via systemic repression of insulin signaling. Cell.

[10] Zhao Q., Wang J., Levichkin I.V., Stasinopoulos S., Ryan M.T., Hoogenraad N.J. (2002). A mitochondrial specific stress response in mammalian cells. EMBO J.

[11] Pellegrino M.W., Nargund A.M., Kirienko N.V., Gillis R., Fiorese C.J., Haynes C.M. (2014). Mitochondrial UPR-regulated innate immunity provides resistance to pathogen infection. Nature.

[12] Gariani K., Menzies K.J., Ryu D. (2016). Eliciting the mitochondrial unfolded protein response by nicotinamide adenine dinucleotide repletion reverses fatty liver disease in mice. Hepatology.

[13] Fiorese C.J., Schulz A.M., Lin Y.F., Rosin N., Pellegrino M.W., Haynes C.M. (2016). The transcription factor ATF5 mediates a mammalian mitochondrial UPR. Curr Biol.

[14] Mouchiroud L., Houtkooper R.H., Moullan N. (2013). The NAD(+)/sirtuin pathway modulates longevity through activation of mitochondrial UPR and FOXO signaling. Cell.

[15] Canto C., Houtkooper R.H., Pirinen E. (2012). The NAD(+) precursor nicotinamide riboside enhances oxidative metabolism and protects against high-fat diet-induced obesity. Cell Metab.

[16] Zhang H., Ryu D., Wu Y. (2016). NAD(+) repletion improves mitochondrial and stem cell function and enhances life span in mice. Science.

[17] Shpilka T., Haynes C.M. (2018). The mitochondrial UPR: mechanisms, physiological functions and implications in ageing. Nat Rev Mol Cell Biol.

[18] Walter P., Ron D. (2011). The unfolded protein response: from stress pathway to homeostatic regulation. Science.

[19] Boulon S., Westman B.J., Hutten S., Boisvert F.M., Lamond A.I. (2010). The nucleolus under stress. Mol Cell.

[20] Gomez-Pastor R., Burchfiel E.T., Thiele D.J. (2018). Regulation of heat shock transcription factors and their roles in physiology and disease. Nat Rev Mol Cell Biol.

[21] Haynes C.M., Petrova K., Benedetti C., Yang Y., Ron D. (2007). ClpP mediates activation of a mitochondrial unfolded protein response in C. elegans. Dev Cell.

[22] Munch C. (2018). The different axes of the mammalian mitochondrial unfolded protein response. BMC Biol.

[23] Horibe T., Hoogenraad N.J. (2007). The chop gene contains an element for the positive regulation of the mitochondrial unfolded protein response. PLoS One.

[24] Nargund A.M., Fiorese C.J., Pellegrino M.W., Deng P., Haynes C.M. (2015). Mitochondrial and nuclear accumulation of the transcription factor ATFS-1 promotes OXPHOS recovery during the UPR(mt). Mol Cell.

[25] Nambi V., Liu X., Chambless L.E. (2013). Troponin T and N-terminal pro-B-type natriuretic peptide: a biomarker approach to predict heart failure risk--the atherosclerosis risk in communities study. Clin Chem.

[26] Hetz C., Chevet E., Harding H.P. (2013). Targeting the unfolded protein response in disease. Nat Rev Drug Discov.

[27] Lin Y.F., Schulz A.M., Pellegrino M.W., Lu Y., Shaham S., Haynes C.M. (2016). Maintenance and propagation of a deleterious mitochondrial genome by the mitochondrial unfolded protein response. Nature.

[28] Martinez B.A., Petersen D.A., Gaeta A.L., Stanley S.P., Caldwell G.A., Caldwell K.A. (2017). Dysregulation of the mitochondrial unfolded protein response induces non-apoptotic dopaminergic neurodegeneration in C. elegans models of Parkinson's disease. J Neurosci.

