# Supplementary material for: Cardioprotective Effect of the Mitochondrial Unfolded Protein Response During Chronic Pressure Overload
Source: J Am Coll Cardiol. 2019 Apr 16;73(14):1795–806. doi: 10.1016/j.jacc.2018.12.087 (PMC6456800; doi:10.1016/j.jacc.2018.12.087)
Supplement: Online Data [file mmc1.docx]

**SUPPLEMENTAL MATERIAL**

**Cardioprotective Effect of the Mitochondrial Unfolded Protein Response During Chronic Pressure Overload**

Smyrnias I, Gray SP, Okonko DO, Sawyer G, Zoccarato A, Catibog N, López B, González A, Ravassa S, Díez J, Shah AM.

**Methods**

***Animal Studies***

Animal procedures were performed in accordance with the Guidance on the Operation of the Animals (Scientific Procedures) Act, 1986 (United Kingdom) and institutional ethical approval. Male mice on a C57BL/6 background were purchased from Envigo (UK). Minimally invasive transverse aortic constriction (TAC) was performed in 8-10 week old mice under 2% isoflurane anesthesia (1). Sham procedures involved identical surgery apart from aortic constriction (SHAM). Echocardiography was performed under 1.5% isoflurane anesthesia using a Visualsonics Vevo 2100 system with a 40-MHz transducer (2).

***Reagents***

Nicotinamide riboside was kindly provided by ChromaDex, Inc USA. The inhibitor of mitochondrial Hsp90, gamitrinib triphenylphosphonium (G-TPP), was kindly provided by D. Altieri (The Wistar Institute Cancer Center, Philadelphia USA). The plasmid expressing Δ-OTC was kindly provided by Dr. Richard J. Youle (Porter Neuroscience Research Centre, Bethesda USA).

***Cell Respiration***

Primary cultures of neonatal rat cardiomyocytes were prepared as previously described (2). Cardiomyocyte oxygen consumption rate was measured on an extracellular flux analyzer (Seahorse), according to the manufacturer’s protocol for cardiomyocyte mitochondrial stress tests. Cardiomyocytes (100,000 cells/well) were cultured on gelatin-coated Seahorse XFe24 culture plates. Basal oxygen consumption rate, oxygen consumption for ATP generation, and maximal oxygen consumption rate were derived from a protocol involving the sequential administration of oligomycin (1 µmol/L), FCCP (2 µmol/L), and rotenone and antimycin A (2 and 4 µmol/L respectively). Basal and FCCP-stimulated oxygen consumption rate were adjusted for non-respiratory oxygen consumption. Data were normalized by the number of cells seeded.

***Mitochondrial function in heart tissue***

Tissue myocardial oxygen consumption rate was measured in an Oxytherm (Hansatech, UK) as previously described (3). Briefly, ventricular muscle fibers were finely dissected in ice-cold relaxing solution and permeabilized with saponin. Measurements were made at 37^o^C. After achievement of steady state basal respiration, glutamate (2 mmol/L) and malate (10 mmol/L) were added to maximize complex I-dependent state 2 respiration. State 3 respiration was stimulated with ADP (5 mmol/L). Cytochrome C (10 µmol/L) was then added to check the integrity of the outer mitochondrial membrane, followed by succinate (10 mmol/L) to maximize complex I+II-dependent oxygen consumption rate. Inhibition of complex I activity with rotenone (0.5 µmol/L) enabled quantification of isolated complex II activity. Finally, maximal uncoupled complex IV activity was measured by adding FCCP (2 μg/ml), antimycin A (2.5 μmol/L), N,N,N,N-tetramethyl- p-phenylenediamine (2 mmol/L), and ascorbate (0.5 mmol/L). Oxygen consumption rate was expressed as nmol O_2_/min/mg of myocardium.

***Atf5 silencing***

Atf5 was silenced in primary rat cardiomyocyte cultures using TransFectin (Bio-Rad, UK)-mediated siRNA transfection. Myocytes were incubated with the transfection mix containing Atf5 siRNA (ThermoFisher, UK) at a final concentration of 150 nM for 12-18 h at 37^o^C. Myocytes were then washed and incubated for at least 48 h before any subsequent treatments or mRNA analysis. Atf5 siRNA sequences were: 5’-3’ sense, GCUAGAACAGAUGGAAGACTT, antisense GUCUUCCAUCUGUUCUAGCTC.

***Real time PCR and immunoblotting***

mRNA levels were quantified by real-time PCR using SYBR Green and the comparative Ct method, with cytoskeletal β-actin levels employed for normalization. Primer sequences are provided in Online Table 1. Immunoblotting of cell or heart lysates was performed using standard methods. The anti-KDEL antibody was from Enzo Life Sciences (UK). Protein bands were quantified by enhanced chemiluminescence or an Odyssey Li-Cor imaging system (Li-Cor Biosciences, UK).

***Histology***

Hearts were arrested in diastole with KCl and fixed with 4% paraformaldehyde as previously described (2). 6 µm transverse cross-sections of paraffin embedded hearts were stained with Terminal Uridine Nick-End Labelling (TUNEL, Millipore S7110 kit) for the assessment of apoptosis.

***Human samples***

The study conformed to the principles of the Declaration of Helsinki and was approved by the pertinent Institutional Ethics Committees. Patients with aortic stenosis were recruited at the Clinic Hospital (Barcelona, Spain) and Virgen de la Victoria University Hospital (Málaga, Spain) and provided informed written consent. The study population consisted of 17 subjects (15 male, 2 female; mean age 73 years) with clinically diagnosed severe isolated aortic stenosis defined in accordance with the following criteria: medium transvalvular pressure gradient (TPG) >40 mmHg, aortic valve area (AVA) *<*1cm^2^ or AVA index *<*0.6cm^2^/m^2^ (4). The patients were referred for valve replacement due to the presence of characteristic clinical symptoms (i.e. angina, syncope, dyspnea or heart failure) and/or LV ejection fraction <50%. Patients with cardiac valve diseases other than AS, those with a history of acute myocardial infarction, those with significant (*>*50%) stenosis in one or more coronary arteries at angiography, or those with disorders associated with alterations in collagen turnover were excluded after a complete medical examination. Blood samples were obtained during admission for aortic valve replacement.

During surgical replacement of the aortic valve, two myocardial samples were obtained from the interventricular septum. The biopsy procedure was well tolerated in all cases and no complications were recorded. One sample was divided into two smaller pieces and frozen separately in liquid nitrogen following different processing procedures for protein and RNA isolation. The other sample was immediately fixed in 4% buffered formalin and embedded in paraffin for histological analyses (the determination of cell apoptosis and collagen quality).

Cell apoptosis was assessed by TUNEL, as previously described (5). Discrimination of TUNEL-positive cells as cardiomyocytes was performed by immunofluorescence for α-sarcomeric actin using a specific mouse monoclonal antibody (1:100 dilution; Sigma) followed by an Alexa Fluor 568-conjugated secondary antibody (1:100 dilution; Invitrogen). The cardiomyocyte apoptotic index was calculated as the percentage of TUNEL-positive cardiomyocyte nuclei with respect to the total number of cardiomyocyte nuclei. To assess the degree of interstitial collagen cross-linking, an enzymatic and colorimetric procedure was used to evaluate insoluble (cross-linked) and soluble collagen, as previously described (6). First, a Fast Green/Sirius Red assay was performed to identify and quantify the total collagen. In a second step, a sircol-based assay was performed to obtain and quantify the soluble collagen. The amount of insoluble collagen was calculated by subtracting the amount of soluble collagen from the amount of total collagen. The degree of interstitial collagen cross-linking was calculated as the ratio between insoluble and soluble collagen. All measurements were performed in duplicate. The inter- and intra-assay coefficients of variation were 5% and 3% respectively.

Plasma N-terminal cleavage fraction (NT)-pro-brain natriuretic peptide (NT-proBNP) was measured using an ELISA (Roche Diagnostics). The lower detection limit of the assay was 5pg/mL and the inter-assay coefficient was 3%. Serum high-sensitivity troponin T was measured using a high-sensitivity assay (Troponin T hs STAT, Roche Diagnostics). The lower detection limit of the assay was 5ng/L and the inter-assay coefficient was 4.6%.

The control LV myocardial samples were obtained from 8 subjects (5 male, 3 female; mean age 44 years) who had died from non-cardiovascular-related diseases, including traumatic brain injury and cerebral hemorrhage.

***Echocardiography***

Parameters assessing LV morphology and function were measured as previously published (7). Mean and maximal TPG were assessed in all patients using the simplified Bernoulli equation, TPG = 4v^2^, where v represents the flow velocity. Aortic valve area index was assessed in all patients according to procedure described by Skjaerpe *et* *al* (8).

***Statistics***

Data are represented as mean±SEM, unless otherwise stated. Comparisons were made by Student’s t test, Mann-Whitney U test or 1-way ANOVA, 2-way ANOVA or repeated measures ANOVA as appropriate, followed by a Bonferroni post-hoc test for multiple comparisons. Human data were analyzed for normality using a Shapiro-Wilk Normality test and subsequently for statistical differences using a non-parametric Mann-Whitney U-Test. P<0.05 was considered significant.

**References**

1. Zhang M, Mongue-Din H, Martin D et al. Both cardiomyocyte and endothelial cell Nox4 mediate protection against hemodynamic overload-induced remodelling. Cardiovasc Res 2018;114:401-408.

2. Smyrnias I, Zhang X, Zhang M et al. Nicotinamide adenine dinucleotide phosphate oxidase-4-dependent upregulation of nuclear factor erythroid-derived 2-like 2 protects the heart during chronic pressure overload. Hypertension 2015;65:547-53.

3. Kuznetsov AV, Veksler V, Gellerich FN, Saks V, Margreiter R, Kunz WS. Analysis of mitochondrial function in situ in permeabilized muscle fibers, tissues and cells. Nat Protoc 2008;3:965-76.

4. Bonow RO, Carabello BA, Chatterjee K et al. 2008 Focused update incorporated into the ACC/AHA 2006 guidelines for the management of patients with valvular heart disease: a report of the American College of Cardiology/American Heart Association Task Force on Practice Guidelines (Writing Committee to Revise the 1998 Guidelines for the Management of Patients With Valvular Heart Disease): endorsed by the Society of Cardiovascular Anesthesiologists, Society for Cardiovascular Angiography and Interventions, and Society of Thoracic Surgeons. Circulation 2008;118:e523-661.

5. Ravassa S, Gonzalez A, Lopez B et al. Upregulation of myocardial Annexin A5 in hypertensive heart disease: association with systolic dysfunction. Eur Heart J 2007;28:2785-91.

6. Lopez B, Querejeta R, Gonzalez A, Beaumont J, Larman M, Diez J. Impact of treatment on myocardial lysyl oxidase expression and collagen cross-linking in patients with heart failure. Hypertension 2009;53:236-42.

7. Beaumont J, Lopez B, Hermida N et al. microRNA-122 down-regulation may play a role in severe myocardial fibrosis in human aortic stenosis through TGF-beta1 up-regulation. Clin Sci (Lond) 2014;126:497-506.

8. Skjaerpe T, Hegrenaes L, Hatle L. Noninvasive estimation of valve area in patients with aortic stenosis by Doppler ultrasound and two-dimensional echocardiography. Circulation 1985;72:810-8.

**Online Table 1: Primer sequences for RT-PCR**

| **Gene** | **Forward (5’-3’)** | **Reverse (5’-3’)** |
| --- | --- | --- |
| **CHOP (rat)** | AGCTGGACACTGTCTCAAAGG | CACCACACCTGAAAGCAGAA |
| **mtDNAj (rat)** | ATCCCAAAGCCAAGGAGAAG | TCACCTCGTCACTCAACACC |
| **ClpP (rat)** | GTGAGGGCAACCTCAAACCA | ACACTTCCTCTGCTGGGCTA |
| **LonP1 (rat)** | GGTTGAGAATGTAGCCCATGA | TCACGATCTCTGCAGTCAGG |
| **Atf5 (rat** | TGTGCATCCGTGTCTAGGTC | CTGACTATGCCAAATAACCCATAA |
| **Hsp10 (rat)** | AGGTGGCATTATGCTTCCAG | TGACAGGCTGAATCTCTCCAC |
| **Hsp60 (rat)** | AAGCTCTTAGCACACTGGTTTTG | GCTGGTTCTTCCTGTTGTCC |
| **Hsp70 (rat)** | CTACAAGGCGGACGA | TAGGACTCGAGCGCATTCTT |
| **Hsp90 (rat)** | TTTCGTGCGTGCTCATTCT | AAGGCAAAGGTTTCGACCTC |
| **CHOP (mouse)** | GCGACAGAGCCAGAATAACA | GCGACAGAGCCAGAATAACA |
| **mtDNAj (mouse)** | AGTCACCCACACAAGCACTG | CCAGCCTCTCGCCTATCC |
| **ClpP (mouse)** | CACAGACATCGCCATCCA | TCCCTCTCCATTGCTGACTC |
| **LonP1 (mouse)** | GGTTGAGAATGTAGCCCATGA | CGATGATATCCCGAATGGTC |
| **Atf5 (mouse)** | TCCGCTCACACCGTCTCT | AAGGCGAAGGTGGAGGAC |
| **Hsp10 (mouse)** | GGCCCGAGTTCAGAGTCC | TGTCAAAGAGCGGAAGAAACTT |
| **Hsp60 (mouse)** | CAGAGCTGGGTCCCTCACT | CTGTGGGTAGTCGAAGCATTT |
| **ClpP (human)** | CCCGTATCATGATCCACCA | AGAGCTGCTTCTTGAGCTTCAT |
| **CHOP (human)** | CCTCCTGGAAATGAAGAGGAAGAA | CTCTGGGAGGTGCTTGTGAC |
| **mtDNAj (human)** | GAGGACGAGACAGATGTGGAG | TCCTGCGGAGCTATCCAT |
| **Hsp60 (human)** | GATGGAGAAGCTCTAAGTACACTCG | GCTGGTTCTTTCTATTGTCACCA |
| **Atf5 (human)** | TTTGCAGTGCGGGAAGAT | AAAATGAACACCCAGTCACCA |

**Online Table 2. Effect of *in* *vivo* treatment with nicotinamide riboside (NR) on UPR^mt^ markers in mouse heart.**

Data are mean ± SEM for mRNA levels (arbitrary units); n=4-6 per group; *P<0.05 vs vehicle-treated animals.

|  | **Vehicle** | **NR** |
| --- | --- | --- |
| **CHOP** | 0.96 ± 0.08 | 1.22 ± 0.11 |
| **ClpP** | 0.97 ± 0.15 | 1.31 ± 0.15* |
| **Atf5** | 1.00 ± 0.01 | 1.38 ± 0.10* |
| **LonP1** | 1.00 ± 0.04 | 1.38 ± 0.11 |
| **mtDNAj** | 0.95 ± 0.04 | 1.31 ± 0.28* |
| **Hsp60** | 0.92 ± 0.06 | 1.53 ± 0.24 |
| **Hsp10** | 0.93 ± 0.05 | 1.46 ± 0.28 |

**Online Table 3. Clinical parameters in patients with aortic stenosis in subgroups A and B.**

BMI, body mass index; NYHA, New York Heart Association functional class; SBP, systolic blood pressure; DBP, diastolic blood pressure; TPG, transvalvular pressure gradient; AVAi, aortic valve area index; LVMI, left ventricular (LV) mass index; LVEDD, LV end-diastolic diameter; LVESD, LV end-systolic diameter; E/A, maximal early (E) to maximal atrial (A) transmitral Doppler diastolic flow velocity ratio; DT, mitral early flow deceleration time; Anti-coag & Anti-plat, anti-coagulants and anti-platelet agents; ACEi & ARB, angiotensin converting enzyme inhibitor and angiotensin II receptor blockers; MR, mineralocorticoid receptor.

|  | **All patients (n=17)** | **Subgroup A**  **(N=9)** | **Subgroup B**  **(N=8)** |
| --- | --- | --- | --- |
| **Age (years)** | 73±1.7 | 72±1.8 | 73±3.1 |
| **Gender (male/female)** | 2/15 | 2/7 | 0/8 |
| **BMI (kg/m^2^)** | 28.3±1.09 | 27.7±1.59 | 29.0±1.53 |
| **Angina (%)** | 47 | 33 | 62.5 |
| **Mean NYHA Class** | 2.23 | 2.44 | 2.00 |
| **Syncope (%)** | 12 | 0 | 25 |
| **History of heart failure (%)** | 41 | 56 | 25 |
| **Hypertension (%)** | 71 | 78 | 62.5 |
| **Atrial fibrillation (%)** | 35 | 44 | 25 |
| **SBP (mmHg)** | 124±4 | 123±5 | 126±7 |
| **DBP (mmHg)** | 68±2 | 67±2 | 59±2 |
| **Mean TPG (mmHg)** | 53±4.2 | 51±6.5 | 55±5.6 |
| **AVAi (cm^2^/m^2^)** | 0.38±0.03 | 0.35±0.05 | 0.42±0.05 |
| **LVMI (kg/m^2^)** | 135.2±9.57 | 143.4±13.1 | 125.93±14.2 |
| **LVEDD (cm)** | 4.26±0.15 | 4.25±0.23 | 4.28±0.21 |
| **LVESD (cm)** | 2.46±0.14 | 2.45±0.23 | 2.48±0.19 |
| **Doppler E/A ratio** | 1.28±0.33 | 1.69±0.58 | 0.80±0.13 |
| **DT (ms)** | 273±25.3 | 259±41.9 | 289±22.7 |
| **Medications (%)**  Anti-coag & Anti-plat  Diuretics  Beta-blockers  Aspirin  ACEi & ARB  MR antagonists  Calcium antagonists | 53  76  35  29  23.5  12  23.5 | 67  78  33  44  22  22  33 | 37.5  75  37.5  12.5  25  0  12.5 |

**Supplementary Figures**

**Supplementary Fig. 1.** Induction of the UPR^mt^ in cardiomyocytes is a process distinct from the endoplasmic reticulum (ER) stress response or the induction of cytosolic chaperones. (*A-B; E-H*) mRNA levels of the cytosolic chaperones Hsp70 and Hsp90 in cardiomyocytes treated with Iso, paraquat or Δ-OTC overexpression. (*C-D*) Protein levels of KDEL sequence-containing markers of ER stress (i.e. calreticulin, Grp78 and Grp79) after treatment with isoproterenol (Iso), paraquat (Par) or tunicamycin (Tunic). Data are mean ± SEM, n=4-7 per group, *P<0.05 vs respective control for changes in mRNA levels. #P<0.05 vs 6h treatment. ^P<0.05 vs 24h treatment.


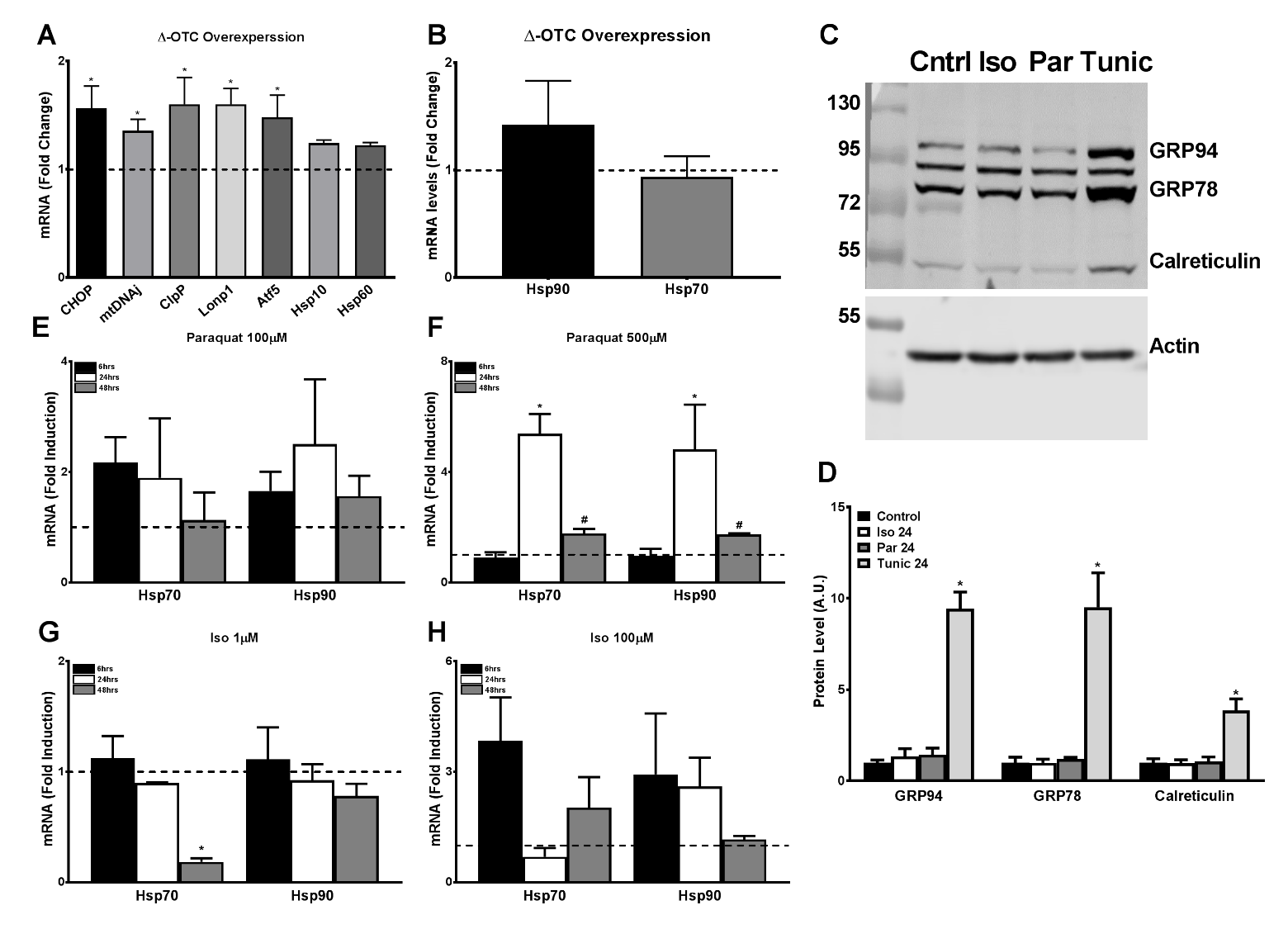


**Supplementary Fig. 2.** Effects of nicotinamide riboside (NR) and olaparib in cardiomyocytes. *(A)* mRNA levels of cytosolic chaperones following treatment with NR. *(B, C)* mRNA levels of UPR^mt^ markers and cytosolic chaperones following treatment with olaparib. *(D, E)* Representative traces (D) and mean data (E) for mitochondrial function and maximal respiration in NRVM treated with isoproterenol and olaparib. Data are mean ± SEM, n=4-6 per group, *^,#^ P<0.05. The dotted line denotes the levels in untreated cells.


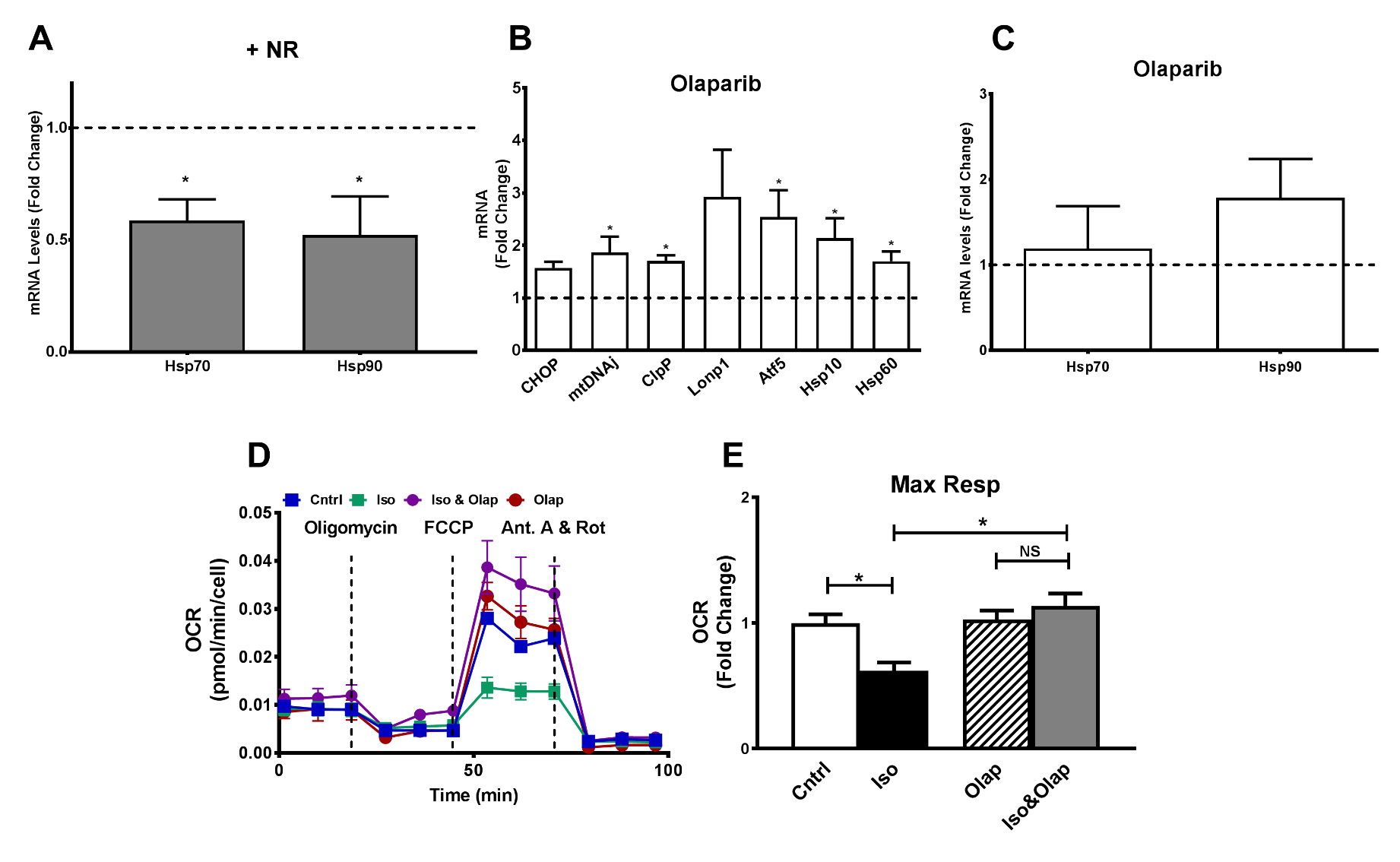


**Supplementary Fig. 3.** Effect of silencing Atf5 on the mRNA levels of UPR^mt^ markers in cardiomyocytes following treatment with Isoproterenol (A) or nicotinamide riboside (NR) (B). *P<0.05 for the comparisons shown.

**
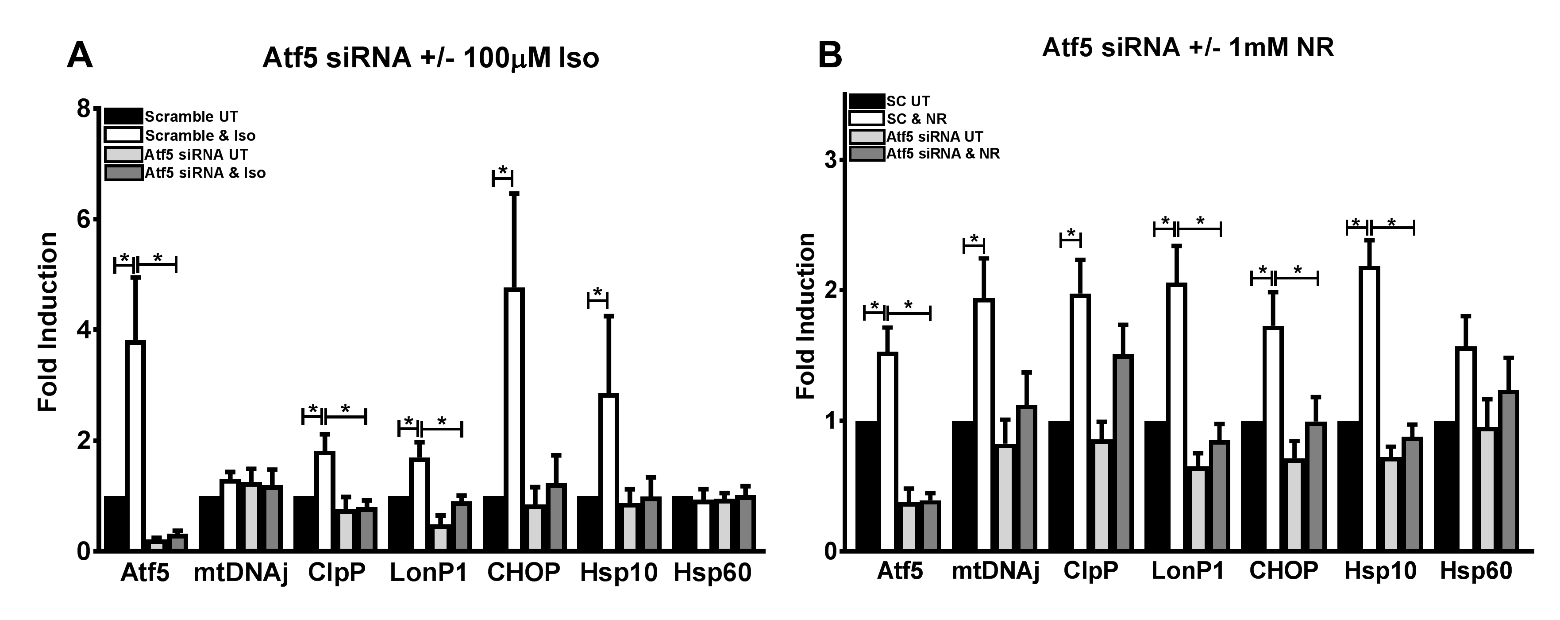
**

**Supplementary Fig. 4.** Effect of treatment with nicotinamide riboside (NR) or vehicle on KDEL sequence-containing proteins in mice subjected to TAC or a sham procedure (Cntrl). n=4 per group.

**
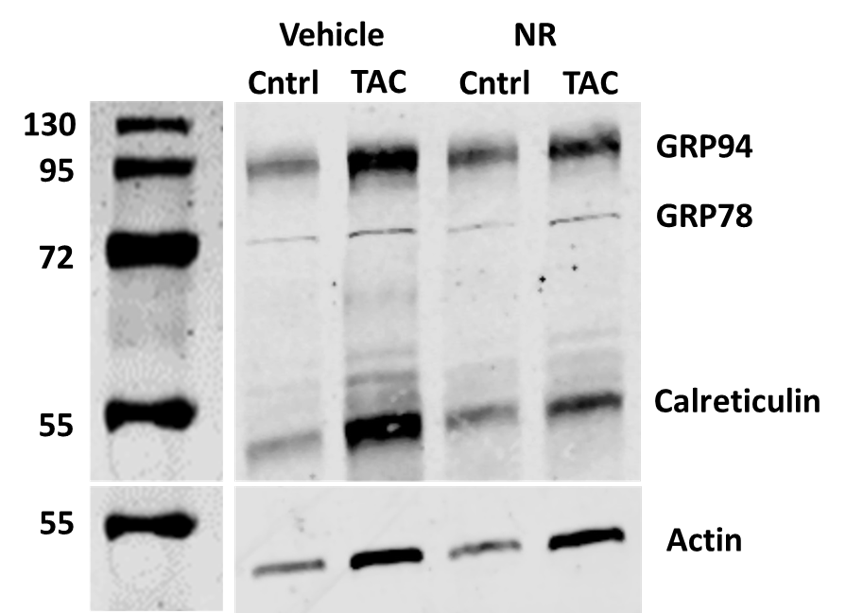
**
